# Role of Tumor-Mediated Dendritic Cell Tolerization in Immune Evasion

**DOI:** 10.3389/fimmu.2019.02876

**Published:** 2019-12-10

**Authors:** Nicholas C. DeVito, Michael P. Plebanek, Bala Theivanthiran, Brent A. Hanks

**Affiliations:** ^1^Division of Medical Oncology, Department of Medicine, Duke Cancer Institute, Durham, NC, United States; ^2^Department of Pharmacology and Cancer Biology, Duke University, Durham, NC, United States

**Keywords:** dendritic cell tolerance, cancer immunotherapy, immune checkpoint inhibition, metastasis, epithelial-to-mesenchymal transition, exosomes, myeloid-derived suppressor cells, dendritic cell immunotherapy

## Abstract

The vast majority of cancer-related deaths are due to metastasis, a process that requires evasion of the host immune system. In addition, a significant percentage of cancer patients do not benefit from our current immunotherapy arsenal due to either primary or secondary immunotherapy resistance. Importantly, select subsets of dendritic cells (DCs) have been shown to be indispensable for generating responses to checkpoint inhibitor immunotherapy. These observations are consistent with the critical role of DCs in antigen cross-presentation and the generation of effective anti-tumor immunity. Therefore, the evolution of efficient tumor-extrinsic mechanisms to modulate DCs is expected to be a potent strategy to escape immunosurveillance and various immunotherapy strategies. Despite this critical role, little is known regarding the methods by which cancers subvert DC function. Herein, we focus on those select mechanisms utilized by developing cancers to co-opt and tolerize local DC populations. We discuss the reported mechanisms utilized by cancers to induce DC tolerization in the tumor microenvironment, describing various parallels between the evolution of these mechanisms and the process of mesenchymal transformation involved in tumorigenesis and metastasis, and we highlight strategies to reverse these mechanisms in order to enhance the efficacy of the currently available checkpoint inhibitor immunotherapies.

## Introduction

During tumorigenesis, the process of malignant transformation occurs concurrently with evasion of the host immune system (1, 2). The ability of tumors to evolve mechanisms to manipulate their local immune microenvironment is also a key component of metastatic progression to distant tissue sites. Given their critical role in orchestrating tumor-targeted immune responses, cancers facilitate their escape from immune recognition and subsequent progression by subverting the functions of antigen presenting cells (APCs) known as dendritic cells (DCs). This process of DC tolerization involves the genetic reprogramming of DCs to ultimately disable immune recognition of developing malignancies (3–6). As the field of immuno-oncology has been primarily focused on directly enhancing the activation of effector T cells, the process of tumor-mediated DC tolerization is comprised of many unexplored opportunities for therapeutically enhancing anti-tumor immunity at earlier stages of the tumor immunity cycle. Herein, we review the processes by which cancers actively drive DC tolerization, how these mechanisms may influence responses to modern immunotherapy, and how these processes can be therapeutically manipulated to improve patient outcomes.

## Dendritic Cell Tolerization in Cancer

DCs represent the functional transition point between the innate and adaptive immune systems and tumor-infiltrating DCs have been described across multiple histologies (7, 8). They have the ability to process antigens derived from the environment and cross-present these antigens to major histocompatibility (MHC) class I-restricted CD8^+^ T cells (9, 10). These DCs further serve to direct the functional programming of the activated T cell, thereby dictating their capacity to defend the host from cancer progression (11, 12).

The phenotypically and functionally distinct subsets of DCs including the plasmacytoid (pDC), conventional (cDC1 or cDC2), and inflammatory DC (moDC), have been extensively reviewed previously (13). Specifically, murine CD8a^+^CD103^+^BATF3^+^CLEC9A^+^XCR1^+^ cDC1s have been demonstrated to have a critical role in the cross-presentation of tumor antigens and are generally thought to be indispensable in the development of host anti-tumor immune responses (14–17). Human cDC1s are necessary for CD8^+^ T cell cross priming and are identified by expression of CD141 (BDCA3) (18) in addition to CD8a, BATF3, XCR1, and CLEC9A (DNGR1) (19–22). In human melanoma samples from the Cancer Genome Atlas (TCGA), the presence of BATF3^+^ DCs was correlated with enhanced CD8^+^ T cell infiltration and T cell homing chemokines CXCL9 and CXCL10 (17). Antigen cross-presentation defects, such as loss of *Batf3, Clec9a*, or *Wdfy4* results in a restrained CD8^+^ T cell repertoire and an inability to reject tumors (23–25). In mouse models lacking BATF3^+^ DCs, IL-12 production and natural killer (NK) cell mediated control of metastasis is impaired while *BATF3* and *IRF8* expression have been associated with improved relapse-free survival in breast cancer patients (26). These data exemplify the importance of DC antigen processing and cross-presentation in the immunologic control of cancer.

**Figure 1:**
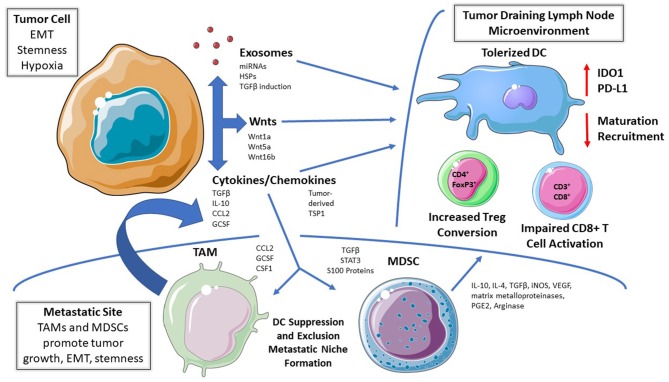
Mechanisms of DC Tolerization in the Tumor Microenvironment. Dendritic cells (DCs) residing within tumor beds, tumor-draining lymph node tissues, or within more distant metastatic sites can be functionally tolerized by tumor-derived soluble mediators, tumor-derived exosomes, and/or via the recruitment of other immunosuppressive cell populations. This process suppresses DC-mediated effector T cell responses while promoting DC-dependent regulatory T cell (Treg) differentiation; thereby facilitating cancer progression and metastasis. EMT, epithelial-mesenchymal transition. TAM, tumor-associated macrophage; MDSC, myeloid-derived suppressor cell; IDO, indoleamine 2,3-dioxygenase; RA, retinoic acid; Arg, arginase; TSP1, thrombospondin-1.

Tumors condition the pre-metastatic niche to develop a favorable immune microenvironment and progressively adapt to immune pressure during dissemination (Figure 1) (27). Therefore, DCs represent logical targets for the evolution of tumor-mediated suppressive mechanisms to facilitate their local and metastatic progression and it is these mechanisms which drive DC tolerization. Despite the advances in our understanding of DC subsets, it remains unclear whether there are unique phenotypic identifiers of tolerized DCs and whether there are multiple subtypes of tolerized DC populations that utilize different modalities to drive immune suppression. To date, investigators have largely utilized the functional conversion of naïve CD4^+^ T cells to the immune suppressive CD4^+^FoxP3^+^ regulatory T cell population (Tregs) coupled with an impaired ability to induce the activation of effector CD8^+^ T cells as their defining features (24, 25, 28).

The recent literature has provided some emerging examples of these immunosuppressive DC subsets contributing to tumor progression and suggests some markers that may identify them. For example, expression of macrophage galactose N-acetyl-galactosamine-specific lectin 2 (MGL2; CD301b; or CLEC10A) was previously described in dermal populations of DCs that promote Th2 differentiation in the draining lymph nodes (29). More recently, in an orthotopic model of pancreatic cancer that metastasizes to the liver, Kenkel et al. described an immunosuppressive subset of hepatic MGL2^+^PD-L2^+^CD11b^+^F4/80^−^ DCs that accumulate in metastatic loci. These DCs promoted Treg development *in vivo* and *in vitro*, and the development of metastasis was hindered by anti-PD-L2 or MGL2^+^ cell depletion (5). In an ovarian cancer model, tumor-driven *Satb1* overexpression in terminally differentiated DCs results in a tolerant, pro-inflammatory state as evidenced by the secretion of Galectin-1 and IL-6, promoting tumor growth and immune evasion (30). Additionally, tumor draining lymph nodes from a Lewis Lung carcinoma model harbor DCs with elevated cyclooxygenase-2 (COX-2) while inhibition of COX-2 results in diminished Tregs and reduced lymph node metastasis suggesting that COX-2 may also promote and be a marker of DC tolerization (31). Experiments performed in a p53-inducible metastatic model of ovarian cancer revealed an MHCII^lo^CD40^lo^PD-L1^hi^ subset of DCs which suppressed CD8^+^ T cell proliferation and failed to induce IFN-γ and Granzyme B production, an effect attributed to TGFβ and prostaglandin E2 (PGE2). The investigators also identified an increasing population of these tolerogenic DCs with metastatic progression and further found that depletion of DCs later in tumor progression using a CD11c-DTR (diphtheria toxin receptor) system impaired tumor growth, suggesting the activation of a phenotypic switch driving DC tolerization during cancer progression (32). Others have also identified tumor-derived PGE2 and TGFβ as being capable of inducing a CD11c^lo^CD11b^hi^ arginase-expressing DC subset which impairs T cell activation, while additional studies have defined a CD11c^hi^CD11b^+^MHC II^+^ DC population that inhibits CD8^+^ T cell responses in several murine tumor models in an arginase-dependent manner (33, 34).

Plasmacytoid DC (pDCs) subsets, defined as CD11c^+^PDCA-1^+^ in mice and CD11c^−^CD123^+^CLEC4C^+^ in humans, have been implicated in the maintenance of peripheral tolerance, as well as the control of anti-viral immunity via the production of type I interferons, exemplifying their functional plasticity (3, 35). pDCs have broadly been associated with poor prognosis across multiple tumor types, perhaps due to their ability to promote Th2 differentiation via the expression of OX40L and ICOSL (3). Further studies have indicated that the more rapid turnover of surface MHC II:Ag complexes on pDCs relative to conventional DCs contributes to their preferential ability to drive Treg development (36). In addition, ovarian cancer-associated pDCs have been characterized as expressing less IFN-α and stimulating higher levels of IL-10-expressing CD4^+^ T cells compared to their circulating counterparts (37). Indeed, the stromal-derived factor-1 (CXCL12) chemokine has been implicated in the recruitment of pDCs to ovarian cancer epithelial tissues to generate an immunosuppressive microenvironment (38). Importantly, Munn and Sharma et al. have described an IDO1-expressing pDC subset in the tumor draining lymph node that is capable of inducing Treg generation, T cell anergy, and potently suppressing T cell response to tumor antigens (39, 40). More work is needed to understand the diversity of pDCs in cancer and to define their individual roles in tumor development, metastasis, and immune regulation.

How these tolerized DC populations are related and how they vary between different tumor types remains unclear. Further studies are warranted to improve our understanding of these associations and determine the functional relevance of these DC markers in human malignancies. In addition, an improved understanding of the relationships between specific oncogenic signaling pathways, the mechanisms driving metastatic progression, and the induction of DC tolerization in the tumor microenvironment promises to ultimately lead to the development of novel strategies for enhancing the efficacy of immunotherapy. Examining the tumor-derived soluble mediators of DC tolerization represents an important step in developing this higher order understanding necessary for translating these findings into clinical trials.

## Soluble Mediators of DC Tolerization in Cancer

### Cytokines and Chemokines

DC development and migration are both significantly altered by paracrine mediators in the microenvironment. Tumors can manipulate this to their advantage to promote metastatic behavior and therapeutic resistance, in part through DC tolerization (Table 1) (56). In one of the earliest reports of tumor-mediated DC tolerization, progressive melanoma tissues refractory to a chemoimmunotherapy regimen were shown to inhibit DC-dependent T cell proliferation via the IL-10 cytokine (57). In a murine breast cancer model, TAMs were the primary source of IL-10 and IL-10R was expressed at high levels on DCs leading to the suppression of the anti-tumor cytokine IL-12. Blockade of IL-10 restored DC function and IL-12 production, and when combined with CSF-1 inhibition, reduced metastatic burden and improved the efficacy of paclitaxel chemotherapy in a manner dependent upon DC production of IL-12 (58). Tumor production of PGE2 impairs recruitment of NK cells responsible for CCL5 and XCL1 production, ultimately reducing intra-tumoral cDC1 migration leading to immune evasion and metastatic progression (59). Other paracrine mediators, such as the release of the tumor cell death factor high-mobility group box-1 (HMGB1), have been shown to bind to TIM-3 on DCs and impair their ability to orchestrate anti-tumor immune responses (33). Various mechanisms leading to β-catenin activation in tumors have also been implicated in the suppression of DC function via enhanced paracrine IL-10 signaling and inhibition of BATF3^+^ CD103^+^ dendritic cell recruitment via CCL4 downregulation (17, 60, 61).

**Table 1:** Tumor-derived Factors Inducing DC Tolerization.

**Tumor derived factor**	**Mechanism of DC tolerance**	**DC marker(s) induced**	**References**
PGE2	↓DC-mediated CD8^+^ T cell proliferation and cytotoxicity	MHCII^lo^CD40^lo^PD-L1^hi^CD11c^lo^CD11b^hi^ Arg^+^	(32)
TGF-β^*^	↓DC-mediated CD8^+^ T cell proliferation and cytotoxicity	MHCII^lo^CD40^lo^PD-L1^hi^ CD11c^lo^CD11b^hi^ Arg^+^	(33, 34)
	↑DC-mediated Treg generation	↓CD86 and CD80, ↓IL-12	(41)
	↑pDC-mediated Treg generation	↑IDO1	(42)
	↓Recruitment of Batf3^+^ DCs	N/A	(43, 44)
Wnt5a	↑DC-mediated Treg generation	β-catenin activation, ↑IDO1,↓IL-12,↓IL-6	(45, 46)
Wnt16b, Wnt1	↑DC-mediated Treg generation	β-catenin activation, ↓CXCL9	(47, 48)
HMGB1	↓DC-mediated CD8^+^ T cell activation	↑TIM3	(33)
CXCL12	↑pDC Recruitment	N/A	(38)
GCSF	↓cDC1 lineage development	↓ IRF8	(6)
CCL2	↑Treg development	HLA-DR, PD-L1	(49)
VEGF	↓DC Maturation	↓MHCII, ↓CD40,↓CD86, ↓IL-12	(50–53)
Tumor-derived Exosomes	Arginase-1 Delivery	↑Arg-1	(54)
	mir-212-3p Delivery	↓MHCII	(55)

* *May also be derived from TAMs, CAFs, MDSCs*.

In addition to tumor cells, other cell populations within the tumor microenvironment also express cytokines and chemokines that influence DC-dependent immunity. Stromal production of immunosuppressive chemokines CCL2, which promote tumor metastasis and M2 macrophage recruitment, have also been described (62, 63). CCL2 has been demonstrated to cooperate with Lipocalin-2 to induce Snail-dependent epithelial-to-mesenchymal transition (EMT) in tumors and to generate immunoregulatory DCs which exhibit decreased levels of HLA-DR expression, upregulated PD-L1 expression, and that functionally induce Treg differentiation (49). Collectively, these findings demonstrate that soluble cytokines and chemokines in the tumor microenvironment play an important role in tumor immune evasion by manipulating DC function.

IFN-γ is also well-known to induce the expression of compensatory regulators, including PD-L1, suppressor of cytokine 2 (SOCS2), and IDO1. Previous studies have described PD-1 and PD-L1 as markers of immunosuppressive DCs that proportionally increase as tumors progress (64, 65). In addition, constitutive IFN-γ signaling in metastatic human melanomas has been associated with an upregulation of the protein suppressor of cytokine signaling-2 (SOCS2) in DCs, which limits their ability to prime T cells, and may serve as a marker of “exhausted” regulatory DCs (66). Finally, granulocyte colony stimulating factor (G-CSF) suppresses cDC1 lineage development via Irf 8, leading to impaired anti-tumor immunity in breast and pancreatic cancer mouse models. Interestingly, fewer relative cDC1 cells in the bone marrow of breast and pancreatic cancer patients have also been associated with poor clinical outcomes (6). The ability of select soluble proteins to promote DC tolerance and contribute to cancer progression have been investigated more extensively and are discussed in the following sections.

### Transforming Growth Factor-β

TGF-β, a paracrine mediator and principal driver of EMT in cancers, has also been implicated in DC tolerance. Co-culture studies of human DCs with lung carcinoma cells resulted in the generation of TGF-β-producing DCs, which exhibit decreased expression of CD86 and IL-12 and an increased ability to generate Tregs (41). TGF-β also promotes the conversion of tumor associated pDCs into a suppressive phenotype by inhibiting IFN-α and MHCI expression in cells activated by the toll like receptor 9 (TLR9) agonist, CpG. Mice lacking pDCs exhibit impaired tumor growth and Treg recruitment, and *in vivo* treatment with anti-TGF-β led to control of tumor growth and diminished recruitment of Tregs (42). Tumor-derived TGF-β suppresses CD80 and CD86 costimulatory molecule expression by DCs and promotes the development of a PD-L1-expressing immunosuppressive DC subset capable of inhibiting CD8^+^ T cell activity in a metastatic ovarian cancer model (32, 33, 67). Loss of the type III TGF-β receptor (TGFβR3) negatively regulates the TGF-β signaling pathway in soluble form following its surface cleavage and suppresses metastatic progression. This process is accompanied by enhanced TGF-β signaling in local DC populations, resulting in IDO1 upregulation in pDCs and CCL22 production in cDCs, both resulting in the accumulation of Tregs and the suppression of anti-tumor immunity (68). These data indicate an overlap between TGFβ-mediated EMT and tumor-associated DC-mediated immunosuppression. TGF-β is known to contribute to an overall immunosuppressive microenvironment, promoting cross-talk in the tumor with pathways of stemness such as Wnt/β-catenin, which is correlated with impaired recruitment of BATF3^+^ DCs (43, 44). Both small and large TGF-β inhibitors are currently being combined with anti-PD-1/anti-PD-L1 antibodies in ongoing clinical trials in a variety of solid tumor types (Table 2).

**Table 2:** Clinical trial protocols that may impact DC tolerization.

**Agent(s)**	**Mechanism of action**	**Registration number**
Bemcentinib	Inhibition of Axl	NCT03184571
Pexidartinib ARRY-382 Cabiralizumab	Inhibition of CSF1R	NCT02777710 NCT02880371 NCT03336216 NCT03599362
M7824	Dual anti-PD-L1 blockade and TGFβ Trap	NCT03620201
Galunisertib	Type I TGFβ receptor serine/threonine kinase inhibitor	NCT02423343
SAR439459	Pan-TGFβ neutralizing antibody	NCT03192345
Regorafenib Ramucirumab Bevacizumab	Inhibition of VEGF (TKI or mAb)	NCT03406871 NCT03712943 NCT02337491 NCT02999295
LGK974 CGX1321 ETC-1922159	Blockade of Wnt Ligand Secretion via PORCN Inhibition	NCT01351103 NCT02675946 NCT02521844
MK-1454	STING agonism	NCT03010176
Epacadostat NLG919 BMS986205	Selective IDO1 inhibitor	NCT03006302 NCT03414229
Indoximod	Tryptophan Mimetic	NCT02073123
APX005M ABBV-927	CD40 agonism	NCT02706353 NCT03123783 NCT03502330 NCT02988960

### Vascular Endothelial Growth Factors

Vascular endothelial growth factors (VEGFs), are abundant in the tumor microenvironment where they play critical roles in angiogenesis, lymphangiogenesis, and metastatic progression. VEGF promotes recruitment of immunosuppressive myeloid cells, impairs cDC maturation, and facilitates a tolerant lymph node microenvironment (50–53). VEGF binds to neuropilin-1 during lipopolysaccharide-dependent DC maturation, resulting in downregulation of MHC II, CD40, and CD86 as well as diminished production of pro-inflammatory cytokines such as IL-12 (69). In a murine ovarian cancer study, tumor-derived β-defensin, in cooperation with VEGF, also recruits a CD34^−^CD8α^−^ MHC-II^lo^CD11c^hi^CD11b^hi^ DC subset which promotes tumor neovascularization and T cell exclusion (70). Given the plethora of therapeutics directed toward VEGF and potential combinatorial opportunities with immunotherapy, a better understanding of the role of VEGF in DC tolerization and the modulation of anti-tumor immunity could be generated based on immune monitoring studies accompanying these clinical trial protocols (Table 2).

### Wnt Ligands

A role for the Wnt-β-catenin signaling pathway in the genetic re-programming involved in DC tolerization was originally described in 2007, where activation of β-catenin was demonstrated to promote IL-10-expressing CD4^+^ T cells and generate tolerance in a model of experimental autoimmune encephalitis (71). Consistent with this, further work revealed that intestinal DCs required β-catenin to express immunosuppressive factors such as IL-10 and TGF-β and drive DC-dependent Treg differentiation in the gut (72). Based on these findings, we hypothesized that tumors may evolve mechanisms for stimulating the activation of the DC β-catenin signaling pathway to generate an immunotolerant microenvironment more conducive to disease progression. This line of investigation led to the discovery that the melanoma-derived WNT5A ligand both promotes the expression and supports the enzymatic activity of IDO1 in local DCs by inducing the synthesis of its required heme prosthetic group, protoporphyrin IX (45, 46, 73). In addition, by promoting β-catenin-dependent fatty acid oxidation in DCs, WNT5A further diminishes IL-6 and IL-12 pro-inflammatory cytokine expression. These alterations culminate in the development and accumulation of Tregs both *in vitro* and *in vivo* and are dominate over other TLR-dependent maturation stimuli. Illustrating the importance of this pathway, these studies also showed that the genetic silencing of *Wnt5a* in melanoma resulted in a significant influx of activated tumor antigen-specific CD8^+^ T cells (46). In addition, the activation of β-catenin in DCs has been associated with the inhibition of antigen cross-presentation via a mechanism dependent upon a mTOR/IL-10 signaling pathway as well as the enhanced synthesis of retinoic acid capable of also promoting DC-dependent Treg differentiation (74–76). Other Wnt ligands such as WNT16B have been demonstrated to promote DC-mediated Treg development *in vitro* while *WNT1* overexpression in human and mouse lung cancers results in cDC1 β-catenin-dependent downregulation of the T cell-recruiting chemokine, CXCL9 (47, 48).

Wnt ligand signaling is perceived as being primarily limited to local and nearby cell populations. Indeed, the ability of many soluble protein-dependent mechanisms to alter distant DC function such as in draining lymph node tissues is more limited. The recent realization that tumor-derived exosomes are capable of genetically altering distant immune cell populations implies that these extracellular vesicles and their molecular cargo are likely to be very important players in DC tolerization and tumor-mediated immune evasion (54, 55, 77).

### Tumor-Derived Exosomes

Over the past decade, a remarkable amount of evidence has emerged demonstrating the role of extracellular vesicles (EVs) in promoting tumor progression and metastasis (78). Exosomes are a sub-class of EVs ranging in size from 30 to 150 nm that primarily function as a vehicle to deliver nucleic acids and proteins (79–81). During tumor progression, tumor cells release exosomes that transit to distant lymphoid tissues and organs where they promote the formation of a tumor supporting microenvironment called the “pre-metastatic niche” (82). Several studies have demonstrated the capacity for exosomes to promote metastasis. For example, in 2011, Hood et al. demonstrated that melanoma exosomes home to the sentinel lymph node where they induce global gene expression changes in the lymph node microenvironment leading to the recruitment and proliferation of tumor cells (83, 84). Additionally, in a landmark study, Peinedo et al. demonstrated that through the delivery of MET tyrosine kinase, melanoma exosomes drive bone marrow progenitor cells toward a phenotype that promotes melanoma metastasis to the lung (85). In pancreatic ductal adenocarcinoma (PDAC) models, PDAC exosomes were found to carry macrophage migration inhibitory factor (MIF), which induces TGF-β signaling in Kupffer cells in the liver resulting in extracellular matrix (ECM) remodeling, a recruitment of bone marrow-derived macrophages and increased metastasis. Importantly, this phenomenon can be inhibited by blocking MIF (86). These pioneering findings were critical to establishing the role of exosomes and other EVs at promoting metastatic progression and immune evasion, however the effects of EVs on DC-mediated T cell activation remains unclear.

Due to the important role of DCs in activating adaptive immune responses, as well as the established capacity for tumor EVs to induce immune suppression, it is logical to anticipate that EVs can function in part by manipulating DC phenotype. Indeed, a recent report has demonstrated that EVs from ovarian cancers transit over long distances to the draining lymph node where they deliver arginase-1 to DCs resulting in a suppression of CD4^+^ and CD8^+^ T cell proliferation (54). In a separate study, Shen et al. report that tumor-derived exosomes induce immune suppression via the delivery of heat shock proteins (HSP72 and HSP105) to DCs leading to increased IL-6 production. IL-6 subsequently led to STAT3 activation and MMP9 expression in tumor cells, enabling increased metastatic invasion (87). In addition to the EV-mediated delivery of protein cargo to DCs, studies have also identified multiple miRNAs that play an important role in DC functions during tumor progression. For example, Ding et al. demonstrated that pancreatic cancer exosomes deliver miR-212-3p to DCs resulting in silencing of the transcription factor regulatory factor-X associated protein (RFXAP), a critical transcription factor for the expression of the MHC II genes (55).

While tumor cells produce a large amount of the EVs in circulation and in the tumor microenvironment during tumor progression, EVs of other cellular origin also can influence the DC phenotype. For example, Mittlebrunn et al. found that T cells can transfer miRNAs to APCs, including DCs, across the immune synapse, which can alter gene expression (88). Additionally, one study demonstrates that Tregs can transfer miRNAs (primarily miR-150-5p and miR-142-3p) to DCs resulting in the induction of a tolerogenic pathway including increased production of IL-10 and decreased IL-6 (89). While the importance of these mechanisms in the context of cancer and other diseases still remains unclear, the ability of EVs to manipulate DC gene expression via the delivery of miRNAs and protein cargo likely has repercussions in cancer immunity.

A large number of soluble mediators that include chemokines and cytokines, developmental and EMT-associated signaling molecules such as the Wnt ligands and TGF-β, as well as tumor manufactured exosomes have been implicated in the processes of DC tolerization and tumor progression. Table 1 summarizes these varied mechanisms and how they modulate DC function. The extracellular nature of these mediators which rely on ligand-receptor interactions, represent a fortuitous area for drug development and potentially biomarker discovery. While numerous, the tumor-derived extracellular factors are likely to constitute only a fraction of the mechanisms leading to DC tolerance. Indeed, other cell populations and biological processes within the tumor microenvironment are also likely to influence DC tolerance.

## Other Components of the Tumor Microenvironment that Drive DC Tolerization

### Myeloid-Derived Suppressor Cells

Myeloid-derived suppressor cells (MDSCs) play pleiotropic roles in cancer cell progression, metastasis, and recurrence while contributing to immunotherapy resistance by shaping the tumor microenvironment and metastatic niche (90–94). MDSCs are generally categorized into two sub-populations, monocytic myeloid suppressor cells (M-MDSCs) and granulocytic or polymorphonuclear MDSCs (PMN-MDSC) (91, 95). In the mouse, M-MDSCs are phenotypically identified as Ly6c^+^Ly6G^−^CD11b^+^ cells and PMN-MDSCs as Ly6G^+^Ly6C^int^CD11b^+^ (95). In humans, M-MDSCs can be identified as CD11b^+^CD14^+^CD15^−^HLA-DR^lo^ and PMN-MDSCs as CD11b^+^CD14^−^CD15^+^HLA-DR^−^ (95). PMN-MDSCs and M-MDSCs are capable of utilizing several different mechanisms of immunosuppression both in the primary tumor bed as well as within distant sites of metastatic disease. This includes the ability of MDSCs to promote tumor growth and metastasis by inhibiting the maturation and antigen presentation function of DCs while secreting immunosuppressive mediators including IL-10, TGF-β, iNOS, VEGF, matrix metalloproteinases, and PGE2 (90, 96–98). In the pre-metastatic niche, MDSCs secrete IL-10 and IL-4, which may prime DCs for tolerance prior to tumor cell seeding (99).

Myeloid progenitors can also be shifted toward MDSC differentiation and away from DCs and macrophages by tumor-derived soluble factors that induce STAT3 activation, leading toward the MDSC phenotype by suppression of protein kinase C βII (100, 101). Tumor-derived S100A9 activates the NF-κB pathway in myeloid cells and suppresses differentiation toward DCs. S100A proteins can also be produced by MDSCs themselves in a STAT3-dependent manner, representing a potential positive feedback loop to suppress the DC lineage in the setting of a malignancy (102, 103). Other groups have also shown that Inhibitor of Differentiation-1 (ID1) is upregulated in DCs by melanoma-derived TGF-β, shunting DCs to differentiate toward an immature MDSC population. ID1 overexpressing bone marrow-derived DCs have also been implicated in the promotion of tumor growth and lung metastasis (104). Additional studies describing the effect of MDSCs on DCs in cancer are necessary to fully clarify the role of MDSCs in immune evasion.

### Structural Components of the Tumor Microenvironment

While less well-described, the ECM may also have a significant role in impairing DC-mediated tumor rejection. Tumor-associated mucins, such as MUC1, promote metastasis formation and interfere with DC function. Mucins can mask TLRs on APCs, and bind to siglecs and galectins on immature DCs, facilitating IL-10 and TGF-β upregulation and reduced IL-12 and costimulatory molecule expression (105). Other ECM components such as Versican (VCAN) correlate with CD8^+^ T cell exclusion and tumor-intrinsic β-catenin nuclear translocation in colorectal cancer, while proteolysis of VCAN into versikine reverses this effect through the recruitment of CD103^+^ MHCII^hi^ BATF3^+^ DCs via IRF8 (106). Additionally, cancer associated fibroblasts (CAFs) are common cells in the tumor microenvironment that can produce the previously discussed suppressive soluble mediators TGF-β, IL-6, VEGFs, as well as express tryptophan 2,3-dioxygenase (TDO) leading to impaired DC maturation, costimulatory molecule expression, and antigen presenting function (107, 108). Furthermore, Cheng et al. have shown that DCs co-cultured with CAFs upregulate IDO expression, downregulate costimulatory molecules, and facilitate Treg generation while exhibiting impaired antigen presentation in an IL-6-STAT3-dependent manner (109). Further study of the structural components within the tumor microenvironment as well as non-tumor and non-immune cells during metastasis may reveal additional therapeutic avenues for understanding and overcoming DC tolerization.

### Tumor EMT and DC Tolerization

The adroit cancer cell invokes developmental pathways of wound healing that lead to mesenchymal transformation or EMT. EMT is a malleable dedifferentiated state during which tumors migrate from their primary site of development to other organs, even co-opting pathways utilized by immune cells for lymphatic trafficking (110–114). In healthy tissues, developmental processes like EMT are not active, however in pathological states like chronic inflammation, wound healing, and cancer, it plays a pivotal role. EMT has been associated with cancer stemness, immune evasion, and therapeutic resistance and is regulated by a network of transcription factors (TF), extrinsic factors such as hypoxia and nutrient deprivation, microRNAs (miRNAs), and long non-coding RNAs (lncRNAs) which contribute to metastatic progression. However, the effects of these specific elements of EMT on immunosuppression, namely local DC populations, is poorly understood (99, 115–117) (Table 3). When the EMT TF Snail was overexpressed in B16F10 mouse melanoma cells, CD4^+^FoxP3^+^ Tregs were generated via MHCII^lo^ IDO-expressing regulatory DCs that developed in response to tumor production of thrombospondin-1 (TSP1). Snail-overexpressing melanomas were resistant to peptide-pulsed DC vaccines while both intra-tumoral Snail-specific siRNA and neutralization of TSP1 restored T cell infiltration (118).

**Table 3:** Tumor intrinsic signaling pathways inducing DC tolerization or suppressing DC recruitment.

**Intrinsic signaling pathway**	**Mechanism of DC tolerance**	**DC marker(s) induced**	**References**
Snail-TSP1	↑DC-mediated Treg generation	↓MHCII, ↑IDO1	(118)
Loss of TβRIII	↑pDC-mediated Treg generation ↑cDC-mediated Treg recruitment	↑IDO1, ↑CCL22	(68)
Intrinsic β-catenin Activation	↓CCL4 ⇒↓BATF3^+^ DC recruitment	n/a	(17, 61)
Tumor stemness	↓antigenicity, ↑ Immunosuppressive Cytokine production (IL-4, IL-10, TGFβ, CXCL12)	n/a	(119, 120)

Cancers both shape and are molded by the myeloid compartment of their microenvironment, and the process of EMT both recruits and is enhanced by tumor-associated macrophages (TAMs) and MDSC populations. During EMT, tumor cells produce CSF1 which recruits TAMs that are able to produce a diverse array of growth factors, facilitating the formation of a metastatic niche (121). TAMs promote tumor progression via stimulation of cancer cell proliferation, as well as through secretion of IL-10 and TGF-β which impair effector T cells and inhibit DC maturation (58, 122). MDSCs also induce EMT via TGF-β and HGF in a mouse model of melanoma, whereby depletion of MDSCs reversed the EMT process (123). Therefore, suppressive myeloid populations are likely to play important roles in EMT and metastatic niche formation. However, it remains unclear how much the process of DC tolerization contributes to these processes.

Tumors may activate well-conserved stem cell pathways along with EMT, allowing for metastatic seeding, immune evasion, and therapeutic resistance (99, 116, 117, 119, 124). Hypoxia-inducible factors including HIF1α in the tumor microenvironment trigger both stemness and EMT programs in the tumor, while impairing DC mediated anti-tumor immunity (125, 126). Additionally, cancer stem cells produce the immunosuppressive cytokines IL-4, IL-10, IL-13, TGF-β, and express higher levels of PD-L1, B7-H3, CD47, and IDO1 (119, 120), enabling these stem cell populations to manipulate DC function. These cancer initiating cells also express CXCR4 and produce its ligand CXCL12, which leads to recruitment of regulatory DCs. These DCs produce CXCL12 themselves, representing a potential feed-forward mechanism where tolerant DCs recruited by cancer stem cells also maintain their stemness (127, 128).

Our understanding of how stem-like, mesenchymal tumor cells interact with and manipulate DC function is in its infancy. Given the tumor-initiating potential of these cells, determining their mechanisms of immune escape could lead to therapeutic strategies capable of suppressing metastatic progression. Understanding the underlying mechanisms involved in these tumor cell-DC and stromal cell-DC interactions will be critical for overcoming resistance to current immunotherapies.

## DC Tolerization and Checkpoint Inhibitor Immunotherapy

While immune checkpoint blockade (ICB) has demonstrated durable efficacy across multiple tumor types, most patients do not respond (129–132). DCs have been shown to be critical for generating responses to anti-PD-1 antibody therapy (17, 133, 134). Indeed, a subset of BATF3^+^ IRF8^+^ cDC1s are not only required for T cell trafficking, but are also necessary for the generation of effector T cell responses to anti-PD-1 therapy (17, 135). Importantly, processes of metastasis co-opted by tumors such as EMT also influence DC maturation, migration, and phenotype, and are associated with ICB resistance in both melanoma and bladder cancer (136, 137). These studies highlight the importance of DCs in ICB and suggest that targeting DCs to reverse tolerogenesis may sensitize previously unresponsive patients to ICB.

Various strategies utilizing DCs to enhance anti-tumor immunity have been attempted but have so far met with limited success in the clinic, highlighting the need for novel approaches. Progress to date on *ex vivo* generated DC-based vaccines is discussed elsewhere (138). Below we will review selected strategies to enhance anti-tumor immunity by indirect or direct reversal of DC tolerization mechanisms *in situ*. Selected clinical trials that deploy these agents to enhance responses to ICB are listed in Table 2.

### Targeting the Tumor

Axl, a Tyro3, Axl, and MerTK family receptor tyrosine kinase implicated in the process of EMT, tumor progression, and metastasis, was found to be upregulated in patients with melanoma who do not respond to ICB (136). In a murine model of ovarian cancer, inhibition of Axl promotes tumor infiltration of CD8^+^ T cell and CD103^+^ DCs associated with an upregulation of the T cell recruiting chemokines CXCL9 and CXCL10. Axl inhibition enhanced anti-PD-1 ab responses, suggesting that Axl may have promise as a therapeutic target (139).

TAMs produce IL-10, suppressing DC production of IL-12, contributing to immune escape and metastatic progression. Pharmacologic modulation of the tumor's ability to recruit TAMs cia CSF1 has shown efficacy preclinically (58). It remains to be seen whether CSF1/CSF1R targeting will be effective in a clinical setting although clinical trials are underway. Other mechanisms for diminishing TAM recruitment by the tumor or re-polarizing TAMs to the M1 phenotype are also being investigated, including antagonism of CCL2 and/or CCL5 or their receptors (140). Recently, Panni et al. demonstrated in a murine pancreatic cancer model that a partial agonist of CD11b^+^ repolarized TAMs and reduced MDSC infiltration while enhancing intratumoral CD103^+^ DC populations, rendering previously resistant murine pancreatic tumors responsive to checkpoint blockade (141). These findings highlight the potential of modulating the myeloid compartment as a therapeutic approach in improving DC-mediated tumor rejection.

### Augmenting the Cytokine and Chemokine Milieu

Manipulating the pro-tumorogenic cytokines and chemokines in the microenvironment also holds promise for sensitizing tumors to ICB. Our group has demonstrated TGF-β to promote IDO1 expression in plasmacytoid DCs, thus facilitating local Treg differentiation within the tumor microenvironment (68). We have further demonstrated that the inhibition of TGF-β enhances anti-CTLA-4 antibody treatment in an autochthonous melanoma model, and that delayed inhibition of TGF-β, but not initial combinatorial therapy, improves anti-PD-1 antibody responses by reversing adaptive resistance (142). TGF-β inhibitor clinical trials are underway (143, 144), and our data indicate these agents could be particularly effective in anti-PD-1 antibody-refractory tumors. As described above, previous studies have shown VEGF to suppress DC maturation. Bevacizumab, an anti-VEGF blocking monoclonal antibody, has been shown to decrease immature myeloid progenitor cells in the peripheral blood of breast, lung, and colorectal cancer patients and enhance IL-12 production (53). Combinations of anti-PD-1 and regorafenib or ramucirumab, both of which target VEGF receptor 2, have shown activity in gastrointestinal malignancies and are proceeding into later stage development (145, 146).

### Targeting the DC

A variety of therapeutic avenues that aim to reverse tumor-induced tolerogenesis or to promote DC licensing and maturation have been pursued, and may ameliorate the shortcomings of DC-based vaccines and/or support the generation of clinical responses to ICB. As we previously discussed, IDO1 is active in regulatory DCs, but is also expressed by other cells including tumors. Preclinical data demonstrated anti-tumor activity with IDO1 inhibition (147–149). However, targeting IDO1 utilizing the selective inhibitor Epacadostat led to disappointing results in combination with pembrolizumab in the Phase III ECHO-301/KEYNOTE-252 trial (150). Potential reasons for this trial's failure are discussed elsewhere (151), however other methods of targeting the Tryptophan (Trp)—Kynurenine (Kyn)—aryl hydrocarbon receptor (AhR) pathway, including the Trp mimetic, Indoximod (152–154), dual IDO1 and tryptophan-2,3-dioxygenase (TDO) inhibition (155), and AhR inhibition (148) are all under investigation. IDO1 has also been reported to be upregulated by both hypoxia and adenosine, which are typical components of the tumor microenvironment encountered by DCs (156, 157). The HIF1a pathway is activated in tolerogenic DCs, and drugs targeting HIF1a have begun to move into the clinical setting (125). While adenosine has been demonstrated to drive a tolerogenic DC phenotype, importantly by the downregulation of IL-12, and has been shown to diminish CD103^+^ DC recruitment in murine models, human data and the effect of adenosine inhibition on DCs in the setting of anti-tumor immunity is yet to be determined (158, 159). CD40 is expressed on APCs including DCs where it interacts with CD40L resulting in the upregulation of co-stimulatory molecules, MHC molecules, and the release of stimulatory cytokines including IL-12. Agonism of CD40 has been shown in preclinical models to enhance both vaccines and anti-PD-1 antibody treatment, and has moved into several clinical trials (160–162). Myeloid development into DCs is impaired by STAT3 signaling, and inhibition of JAK2/STAT3 has been shown to enhance anti-tumor responses through the promotion of DC maturation in preclinical models (101).

Finally, we have shown that inhibition of the Wnt/β-catenin pathway using an anti-Fzd receptor antibody or a Wnt ligand trap enhances anti-tumor immunity in autochthonous melanoma and Lewis lung carcinoma mouse models. These agents suppressed primary tumor growth and the formation of lung metastasis, and led to improved antigen-specific T cell responses over anti-PD-1 antibody treatment alone (163). We have further demonstrated that small molecule inhibitors of the PORCN acyltransferase enzyme, which effectively block Wnt ligand release, synergistically enhances the efficacy of anti-CTLA-4 antibody immunotherapy in pre-clinical models of melanoma. Others have also shown that deletion or pharmacologic inhibition of the Fzd co-receptors, LRP5/6, in DCs promoted their anti-tumor effects, further highlighting the therapeutic potential of targeting this pathway and a possible method of enhancing DC-based vaccines (164). Clinical trials examining the PORCN inhibitors in combination with anti-PD-1 antibody checkpoint inhibitor therapy are currently ongoing.

### Other Strategies for Reversing DC Tolerance

A myriad of other approaches are also early in therapeutic development. Blockade of the CXCL12/CXCR4 axis utilizing an oncolytic viral vector or small molecule inhibitor impaired tumor stemness and enhanced DC activation (127, 128). Effects of these approaches on metastasis remains unclear, however. Type 1 interferons have been shown to be essential in murine models for DC-mediated anti-tumor immunity and can be induced through several mechanisms including toll-like receptor (TLR) and Stimulator of Interferon Genes (STING) agonists, both of which can impact DC maturation (165). TLRs bind bacterial cell wall components as well as danger-associated molecular patterns, CpG motifs, and ssRNA or dsRNA released during cell death, while the STING pathway is activated in response to cytosolic DNA. In addition to inducing Type 1 IFNs, co-stimulatory molecules (CD80, CD86, CD80) are also upregulated and targeting these pathways may assist in overcoming tumor-induced DC tolerance, particularly when designing therapies that manipulate the DC directly such as vaccines (166). This is exemplified in the development of CDX-1401 which contains a DC receptor (DEC-205, CD205) specific monoclonal antibody to deliver the conjugated tumor antigen NY-ESO-1 in combination with TLR7/8 agonists (167), and is now in clinical trials combined with IDO1 inhibition (NCT02166905). Other groups, in both mouse models and patients, have utilized radiation to release tumor antigens combined with a TLR3 agonist and Flt3L to expose DCs to antigen and foster DC maturation, resulting in immune-mediated tumor elimination at distant sites (known as the abscopal effect) (168). *In situ* DC targeting utilizing viral vectors can potentially provide tumor antigens, co-stimulatory, and maturation (i.e., Flt3L) signals (169), enabling DCs to overcome tumor-induced tolerance. Owing to their complexity, the development of viral vectors and other *in situ* methods to specifically target DCs are likely to require significant time before clinical outcomes are demonstrated (170). Other unique strategies, such as loading DCs with cancer stem cell lysates and the implantation of TLR 7/8 or STING agonists post-operatively to convert the surgical bed into an anti-tumor microenvironment have also been investigated (171–173).

Effectively reversing DC tolerization will likely require therapeutic approaches tailored to the individual tumor type, if not the patient. An improved understanding of tumor-induced DC tolerization mechanisms promises to streamline the selection of more novel, higher yield approaches for the development of future combinatorial immunotherapy strategies.

## Conclusions, Future Directions, and Remaining Key Questions

Herein, we have described those studies implicating an important role for DC tolerization in tumor-mediated immune evasion and immunotherapy resistance. While the field of tumor immunology has made significant advances in the clinic, the majority of our cancer patients still do not benefit from immunotherapy. A significant fraction of the ongoing effort to maintain this momentum in immuno-oncology remains focused on pharmacological and/or genetic manipulation of the effector phase of the anti-tumor immune response. However, we believe that it will be those approaches that effectively combine these strategies targeting cytolytic T cell function in the effector phase with those strategies designed to modulate DC functionality in the priming phase that will ultimately generate clinically meaningful responses in a broader population of cancer patients. Several critical unanswered question relevant to this area remain (Box 1). Technological advancements in single cell technologies promise to help identify populations of tolerogenic and tumor-promoting DCs, elucidating their defining features and perhaps therapeutic targets. We believe this understanding will be enhanced by a renewed focus on the tumor draining lymph node microenvironment and how tumors condition DCs within these tissues to induce immune tolerance. Collaborations between clinicians, translational investigators, and basic scientists will be critical in obtaining patient specimens in order to build upon the progress and promise of immunotherapy—to prevent and treat metastatic cancer, prolonging the lives of those affected.

Box 1 **Unanswered questions about the role of tumor-mediated dendritic cell tolerance during immune evasion.**

What dendritic cell markers define a tolerized DC and are there markers that define more nuanced phenotypes by the functional mechanism of suppression?

How do tumors suppress DC-mediated antigen cross-presentation and do these mechanisms vary by cancer type?

What are the tumor-intrinsic metastasis-initiating events that lead to DC tolerance in both the tumor microenvironment and the draining lymph node?

What role do tumor-derived exosomes play in the induction of DC tolerance, and can they be used as biomarkers and/or as therapeutic vectors?

What role do DCs play in resistance to immune checkpoint blockade and how can we modulate these DCs to enhance current immunotherapeutic strategies in a patient-specific manner?

What strategies will be most effective for modulating DC function in vivo?

## Author's Note

The figure was produced using Servier Medical Art.

## Author Contributions

ND and BH conceptualized the project. ND, MP, BT, and BH wrote and edited the manuscript and approved the final draft for publication.

### Conflict of Interest

BH receives research funding from Merck & Co., Tempest Therapeutics, Leap Therapeutics, A*STAR Singapore, and Astrazeneca. The remaining authors declare that the research was conducted in the absence of any commercial or financial relationships that could be construed as a potential conflict of interest.
